# Direct Z-scheme GaN/WSe_2_ heterostructure for enhanced photocatalytic water splitting under visible spectrum†

**DOI:** 10.1039/d3ra00928a

**Published:** 2023-07-04

**Authors:** Xiaojun Ye, Fangfang Zhuang, Yuhan Si, Jingwen He, Yifan Xue, Hongbo Li, Kai Wang, Guoqiang Hao, Rui Zhang

**Affiliations:** a School of Materials Science and Engineering, East China University of Science and Technology Shanghai 200237 China zhangrui118@ecust.edu.cn; b Shanghai Institute of Space Power-sources, State Key Laboratory of Space Power-sources Technology Shanghai 200245 China haoguoqiang@ecust.edu.cn

## Abstract

van der Waals heterostructures are widely used in the field of photocatalysis due to the fact that their properties can be regulated *via* an external electric field, strain engineering, interface rotation, alloying, doping, *etc.* to promote the capacity of discrete photogenerated carriers. Herein, we fabricated an innovative heterostructure by piling monolayer GaN on isolated WSe_2_. Subsequently, a first principles calculation based on density functional theory was performed to verify the two-dimensional GaN/WSe_2_ heterostructure and explore its interface stability, electronic property, carrier mobility and photocatalytic performance. The results demonstrated that the GaN/WSe_2_ heterostructure has a direct Z-type band arrangement and possesses a bandgap of 1.66 eV. The built-in electric field is caused by the transfer of positive charge between the WSe_2_ layers to the GaN layer, directly leading to the segregation of photogenerated electron–hole pairs. The GaN/WSe_2_ heterostructure has high carrier mobility, which is conducive to the transmission of photogenerated carriers. Furthermore, the Gibbs free energy changes to a negative value and declines continuously during the water splitting reaction into oxygen without supplementary overpotential in a neural environment, satisfying the thermodynamic demands of water splitting. These findings verify the enhanced photocatalytic water splitting under visible light and can be used as the theoretical basis for the practical application of GaN/WSe_2_ heterostructures.

## Abstract 2

van der Waals heterostructures are widely used in the field of photocatalysis due to the fact that their properties can be regulated to promote the capacity of discrete photogenerated carriers.

## Graphical abstract

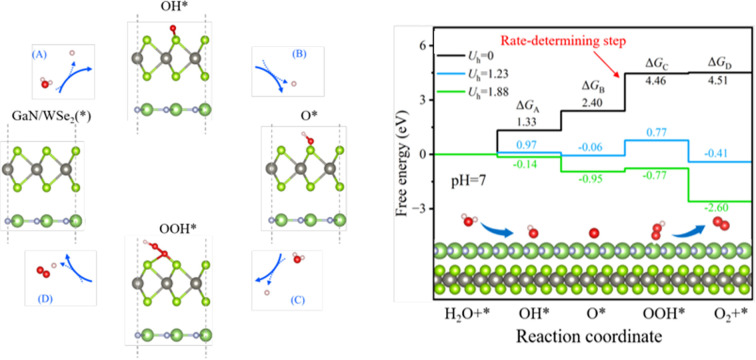

## 1. Introduction

Hydrogen energy is anticipated to be an alternative fuel to fossil fuels in the near future,^1–3^ and in this case, photocatalytic water splitting is an attractive approach to produce hydrogen.^4^ The traditional photocatalytic material TiO_2_ has a wide band gap (BG) of 3.20 eV, which is only activated by ultraviolet light with a wavelength of less than 385 nm and its hydrogen production efficiency is extremely low.^5^ Alternatively, two-dimensional (2D) transition metal dichalcogenides (TMDCs) such as WSe_2_, MoS_2_, and MoSe_2_ have significant application prospects in photocatalytic water splitting due to their excellent electronic properties, high carrier mobilities and visible-light response. In particular, it has been confirmed theoretically that monolayer WSe_2_ has robust photoluminescence and high carrier mobility (705 cm^2^ V^−1^ s^−1^), which are superior to that of MoS_2_ and MoSe_2_.^6–8^ Nevertheless, the photocatalytic performance of monolayer WSe_2_ is still limited owing to its high transmittance and poor photogenerated electron–hole separation efficiency. Hence, the use of a co-catalyst to reduce the electron–hole recombination is strongly recommended.

In recent years, some studies have proven that the construction of van der Waals heterostructures (vdWHs) is a valid approach to improve the photocatalytic efficiency of 2D materials.^9,10^ vdWHs retain the electronic properties of individual layers and the interface effect of heterostructures endow them with some properties that are not present in their respective components, such as regulating the bandgap energy and improving the segregation efficiency of photogenic electron–hole pairs.^11,12^ Traditional type II heterostructures have the advantages of response in an expanded spectrum range and promoted carrier separation,^13^ but their redox ability is poor. Thus, the design and fabrication of novel direct Z-scheme photocatalysts are attracting increasing interest to improve the redox ability and transfer performance of photo-generated charges.^14–16^

GaN monolayers are very promising for application in high-performance opto-electronic devices due to their semiconducting character with a suitable bandgap of about 2.3 eV,^17,18^ which is narrower than that of bulk GaN.^19^ Previous research disclosed that GaN shares an identical hexagonal configuration and related lattice constants with TMDCs, making them compatible.^20^ Also, a GaN thin film was prepared *via* chemical vapor deposition from SL-WSe_2_/c-sapphire to achieve GaN/WSe_2_ heterostructures.^21^ R. Meng *et al.* revealed that the existence of band offsets and intrinsic electric fields leads to reinforced photocatalytic activity between WSe_2_/GaN and WS_2_/GaN.^22^ Shaoqian Yin *et al.* studied the effects of modifying the electric field and strain on the optical and electronic characteristics of GaN/WSe_2_ heterostructures with various stacking configurations. However, a systematic study has not been performed to date on the photocatalytic performance of GaN/WSe_2_ heterostructures.^23^

In this study, the structural stability, electronic properties, carrier mobilities, and photocatalytic performance of GaN/WSe_2_ heterostructures were explored *via* first-principles calculations. The calculations of the energy band gap presented that the GaN/WSe_2_ heterostructure is a representative direct Z-scheme with a built-in electric field from GaN to WSe_2_. Meantime, the carrier mobilities of the GaN/WSe_2_ heterostructure, which influence the dissociation efficiency, was also amplified. In addition, the calculation of the Gibbs free energy of the GaN/WSe_2_ system clarify the oxygen evolution reaction (OER) process. Consequently, it was inferred that GaN/WSe_2_ heterostructures, which possess superior photocatalytic capacities under visible light, are favorable photocatalysts in the field water splitting.

## 2. Calculation methods and models

The theoretical analyses were manipulated entirely through the Vienna *ab initio* simulation package (VASP)^24^ within the projector augmented plane-wave (PAW) pseudopotentials using density functional theory (DFT). The Perdew–Burke–Ernzerhof (PBE) algorithm was adopted to confirm the exchange-correlation functional.^25^ Two-dispersion correction of DFT-TS^26^ and DFT-D3 (ref. 27) was considered in the computation for clarifying the impacts of non-covalent forces. The valence electron schemes were as follows: 3d^10^4s^2^4p^1^ for Ga, 2s^2^2p^3^ for N, 5d^4^6s^2^ for W, and 4s^2^4p^4^ for Se. A Monkhorst Pack *k*-point grid of 7 × 7 × 1 and a cutoff energy of 480 eV were employed in the first Brillouin zone. The maximum force was set as 0.01 eV Å^−1^ and the energy convergence threshold was 10^−5^ eV.

**Fig. 1:**
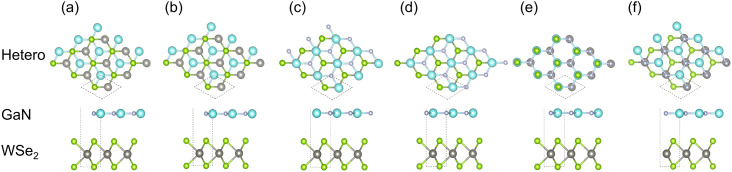
Stereo matching patterns of six canonical parallel configurations of GaN/WSe_2_ heterostructures. (a) model I, where the N atoms in interface are on the Se atoms straightly; (b) model II, where the Ga atoms are located directly above the middle of the W–Se bonds; (c) model III, where the N atoms in the interface are straightly on top the middle of the W–Se bonds; (d) model IV, where the Ga atoms are located directly above the W atoms; (e) model V, where Ga atoms are located directly on the Se atoms; and (f) model VI, where the N atoms are set on the W atoms.

The space group of *P*6/*m*2 was chosen for isolated GaN and WSe_2_. The lattice constants of the structure-optimized GaN (*a* = *b* = 3.200 Å, *γ* = 120°, see Table 1) and WSe_2_ monolayer (*a* = *b* = 3.212 Å, *γ* = 120°, see Table 1) were identical to the anterior empirical and theoretical results.^28–30^ The GaN/WSe_2_ heterostructure was configured by the relaxed 2 × 2 lateral periodicity of monolayer GaN(001) and WSe_2_(001), as shown in Fig. 1(c)–(e). To avoid interlayer interactions, a vacuum spacing of 15 Å was adopted perpendicularly for the GaN/WSe_2_ heterostructures.

**Table 1:** Calculated lattice parameters (Å), lattice mismatch ratio (%), cohesive energies (meV Å^−2^), mismatch energies (meV Å^−2^), vdW binding energies (meV Å^−2^) and equilibrium interlayer distances (Å) of GaN/WSe_2_ heterostructures using the dispersion-correction DFT-TS and DFT-D3 approaches after geometric relaxation

Model	Method	GaN	WSe_2_	GaN/WSe_2_	*R*_mis_	*E*_coh_	*E*_mis_	*E*_vdw_	*d*
		*a*_1_ = *b*_1_	*a*_2_ = *b*_2_	*a* = *b*					
V	DFT-D3	3.200	3.296	3.246	3.00	1.84	−0.78	2.61	3.09
VI	DFT-TS	3.212	3.338	3.261	3.92	4.14	−2.55	6.69	3.32

## 3. Results and discussion

### 3.1 Interface stability

**Fig. 2:**
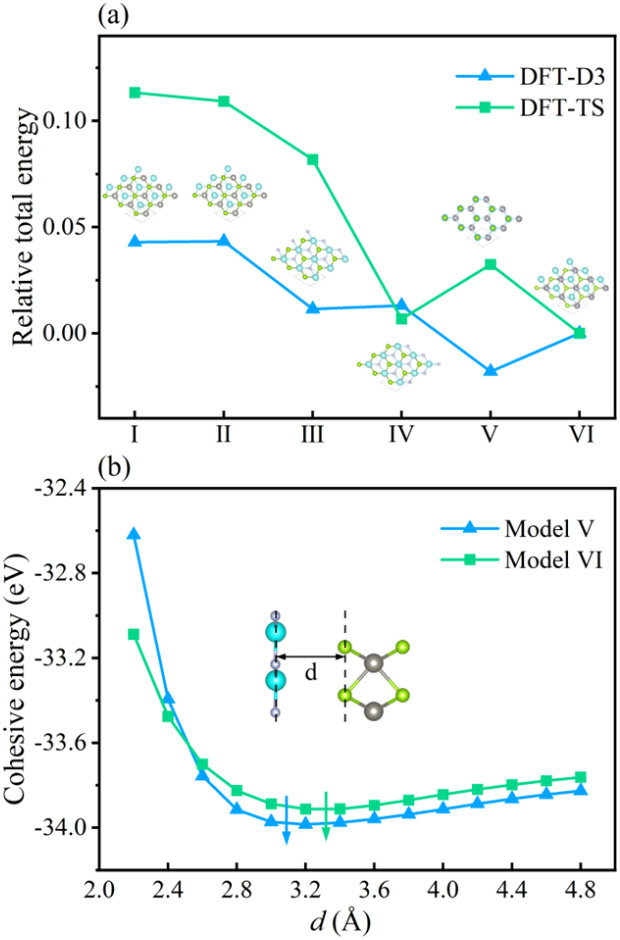
(a) Relative total energies of six models for GaN/WSe_2_ heterostructures with DFT-TS and DFT-D3 approaches after geometrical optimization, where the illustration exhibits the corresponding side view of the heterostructure. (b) Dependence of the cohesive energies of model V and model VI with interlayer distance *d*.

To inspect the impact of the interfacial interactions between the GaN and WSe_2_ nanosheets on the structural stability of GaN/WSe_2_ combinations, the compositional dependence of the total energy is discussed. Six representative parallel alignments of GaN/WSe_2_ heterostructures are displayed in Fig. 1. To calculate the structural stability of the six stacked models quantitatively, the D3 and TS dispersion corrections were included to calculate the relative total energies of the six patterns (compared with the steadiest model) applying the PBE method, respectively, as shown in Fig. 2. The results show that the different calculation methods have analogous variation tendencies in the related total energies of the six configurations, demonstrating that the computational outcomes are dependable. Also, the figure shows that model V has the lowest relative energy among the models with the DFT-D3 algorithm, while model VI has the lowest relative energy compared with the others with DFT-TS algorithm. Hence, all sequent calculations were built on these two models.

To further study the structural stability of the GaN/WSe_2_ heterostructure, the lattice mismatch ratio and mismatch energy between the two monolayers were calculated. The lattice mismatch ratio is described as *R*_mis_=(*a*_2_ − *a*_1_)/*a*_1_, where *a*_1_ and *a*_2_ represent the lattice constants of the GaN and WSe_2_ monolayers, respectively. Table 1 lists the *R*_mis_ of the GaN/WSe_2_ heterostructures calculated by the DFT-D3 and DFT-TS methods, which are 3.00% and 3.92%, respectively, showing a perfect match.^31^ Moreover, the lattice mismatch energies of the GaN/WSe_2_ heterostructures were determined using the following equation:

1 Δ*E*_mis_ = [*E*_(GaN)a_ + *E*_(WSe_2_)a_ − *E*_(GaN)_ − *E*_(WSe_2_)_]/*S*

where *E*_(GaN)*a*_ and *E*_(WSe_2_)*a*_ are the overall energies of isolated GaN and WSe_2_ under the equilibrium lattice parameters of the GaN/WSe_2_ heterostructures, respectively. *E*_GaN_ and *E*_WSe_2__ indicate the whole energies of the single GaN and WSe_2_ nanosheets before contact. *S* represents the interfacial area of the heterostructure. The mismatch energy results are exhibited in Table 1. Δ*E*_mis_ computed by DFT-D3 and DFT-TS approaches is 1.84 meV Å^−2^ and 4.14 meV Å^−2^, respectively, which is substantially below that of WS_2_/WSe_2_,^34^ MoS_2_/WSe_2_,^33^ and graphene/WSe_2_.^32^ In the heterostructure, the lattice mismatch of the isolated GaN and WSe_2_ caused by strain-driven interactions is almost negligible.

To elucidate the adsorption interaction between the GaN and WSe_2_ monolayers, the two above-mentioned methods were chosen to attain the interfacial cohesive energy, *E*_coh_, at different interlayer distances, *d*. *E*_coh_ is expressed as follows:

2 *E*_coh_ = (*E*_GaN/WSe_2__ − *E*_GaN_ − *E*_WSe_2__)/*S*

where *E*_GaN/WSe_2__, *E*_GaN_, and *E*_WSe_2__ correspond to the whole energies of relaxed GaN/WSe_2_ heterostructure, GaN monolayer and WSe_2_ monolayer, separately. The *E*_coh_ of the GaN/WSe_2_ composites at the most stable interlayer spacing is −0.78 meV Å^−2^ and −2.55 meV Å^−2^ for DFT-D3 and DFT-TS, respectively, as displayed in Table 1. It is noticeable that the negative *E*_coh_ values imply stable heterostructures.^35,36^ Therefore, the cohesion between GaN and WSe_2_ stabilizes the geometry. In addition, the *E*_coh_ trends calculated using the two methods are very similar to the changes in the interfacial space, proving the dependability of the calculated results.

Finally, the van der Waals energy was introduced to quantitatively describe the magnitude of the interlayer van der Waals force, which is defined as and its magnitude is determined by the lattice mismatch energy and the interface binding energy. The calculation formula is as follows:

3 *E*_vdW_ = |*E*_coh_| + |Δ*E*_mis_|

The calculated results are 2.61 eV and 6.69 eV, which are within the normal vdW binding energy range,^37,38^ indicating the existence of slight van der Waals forces in the GaN/WSe_2_ heterostructures.

In the case of the GaN/WSe_2_ heterostructure, the equilibrium interfacial space between GaN and WSe_2_ is 3.09 Å with DET-D3 and 3.32 Å with DFT-TS, respectively, which is the canonical distance of vdW force.^39^ Besides, given that *R*_mis_ under DET-D3 is smaller than that with DET-TS, the subsequent calculations are based on model V of the GaN/WSe_2_ heterostructure with the DET-D3 method. The results of model VI of the GaN/WSe_2_ heterostructure with the DET-TS method are demonstrated in the ESI.†

### 3.2 Electronic property

**Fig. 3:**
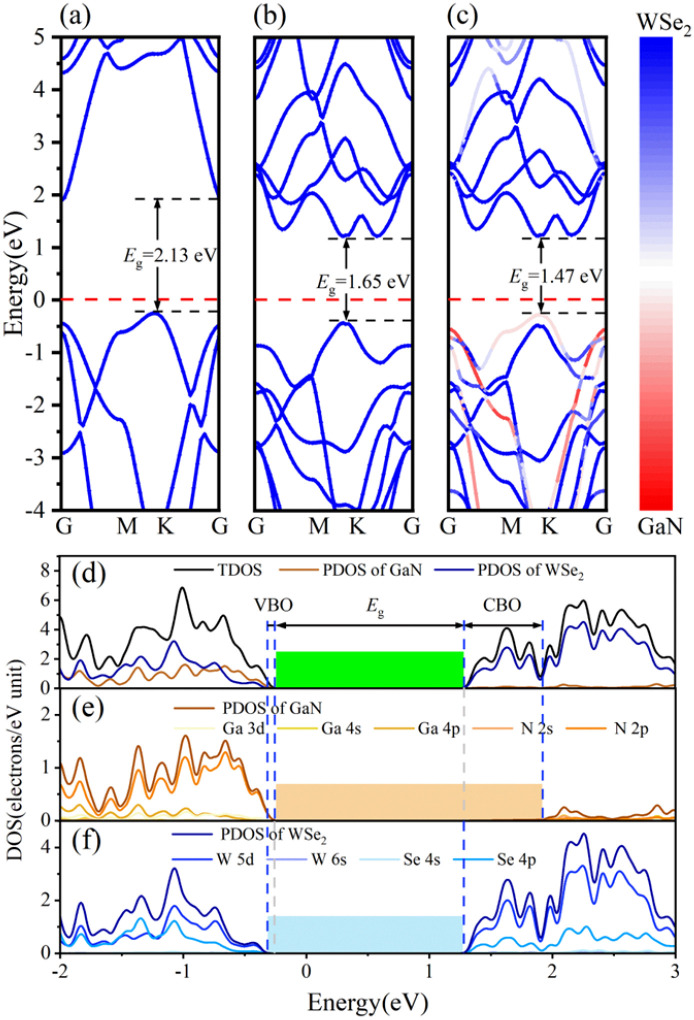
Energy band diagrams of (a) monolayer GaN, (b) WSe_2_ nanosheet, and (c) model V of GaN/WSe_2_ heterostructure. (d) Calculated TDOS of GaN/WSe_2_ heterostructure and PDOS of GaN and WSe_2_ employing the PBE algorithm. (e) PDOS of Ga and N atoms in GaN. (f) PDOS of W and Se atoms in WSe_2_.

The band arrangements of the GaN monolayer, WSe_2_ monolayer and GaN/WSe_2_ composites were investigated using the PBE functional module. The Fermi level of the three systems was set as the level zero energy, and the high symmetry sites G (0,0,0), M (0,0.5,0) and K (−0.333,0.667,0) in the Brillouin zone were used as observation routes to study the band alignment of the system with the range limited from −4 eV to 5 eV. Fig. 3(a) shows that monolayer GaN is a semiconductor with an indirect BG of 2.13 eV, where the valence band maximum (VBM) is settled at the *K* point, while the conduction band minimum (CBM) is settled at the *G* point. This is theoretically in accordance with the established research.^40^ The VBM and CBM of the single-layer WSe_2_ were both set near the *K* point, which denotes that WSe_2_ is a semiconductor with a direct BG of 1.65 eV. The calculated results of WSe_2_ are similar to that reported by R. S. Meng,^41^ as demonstrated in Fig. 3(b).

Fig. 3(c) presents the band diagram of the GaN/WSe_2_ heterostructure. Both the VBM and CBM are located at the high symmetry point *K*, and thus it is a direct semiconductor with a BG of 1.47 eV. Compared with the indirect BG semiconductor, the direct bandgap semiconductor has a higher absorption coefficient for photo-generated electron–hole pairs and higher light utilization rate, and thus it is more suitable for photocatalysis.^42^ The forbidden bandwidth of the GaN/WSe_2_ heterostructure is 1.47 eV, which is slightly lower than that of the GaN and WSe_2_ monolayers, and its energy levels are denser. This is conducive to the migration of electrons, thereby improving the photocatalytic activity. Also, the BG of the GaN/WSe_2_ heterostructure is larger than that required for photocatalytic water splitting, which is 1.23 eV. In addition, comparing the three images in Fig. 3(a–c), it can be found that the energy band structure of the GaN/WSe_2_ heterostructure is similar to the energy band alignments of the two monolayers, and almost retains that of GaN and WSe_2_ to a large extent. Therefore, it can be speculated that the binding force between the heterostructure layers is weak van der Waals force, and the interaction force when the two monolayers are combined is small, which also corresponds with the above-mentioned result.

The density of states (DOS) usually reflects the distribution of electrons in each system. To further elucidate the electronic structure of the GaN/WSe_2_ heterostructure, we also calculated the density of states of the GaN/WSe_2_ heterostructure, GaN monolayer and WSe_2_ monolayer. The density curve range was −2 eV to 3 eV for analysis, as illustrated in Fig. 3(e and f). The VBM of monolayer GaN consists mainly of Ga-4p and N-2p states, whereas the CBM consists of N-2p states, are displayed in Fig. 3(e). The VBM and CBM of monolayer WSe_2_ consist primarily of W-5d and Se-4p states, as presented in Fig. 3(f). Apparently, the VBM is derived from GaN (orange shaded region in Fig. 3(e)), while the CBM of the GaN/WSe_2_ heterostructure originates from WSe_2_ (blue shaded region in Fig. 3(f)). The overlap may be caused by orbital hybridization, and the occurrence of orbital overlap will result in a reduction in the BG, which is more advantageous to improve the catalytic performance of the photocatalytic material.^43^ It is well-known that the valence band offset (VBO) and the conduction band offset (CBO) are at the related sites of the VBM and the CBM of two sides of the interfacial space, respectively, which can considerably modify the charge transfer capacity of the heterostructures under illumination.^44^ In addition, VBO, CBO, and BG can be defined as Δ*E*_V_ = *E*_GaN VBM_ − *E*_WSe_2_ V_, Δ*E*_C_ = *E*_GaN CBM_ − *E*_WSe2 CBM_, and *E*_g_ = *E*WSe_2_ CBM − *E*GaN VBM, respectively. Under the equiponderant interfacial condition, the correlation of VBO, CBO, the BG values is VBO (0.12 eV) < CBO (0.65 eV) < BG (1.47 eV). Accordingly, it can be reliably inferred that the GaN/WSe_2_ heterostructure shows a type-II or Z-scheme band structure, which is consistent with the conclusion of band alignment, leading to the spatial segregation of the photogenerated carrier pairs.^45^

**Fig. 4:**
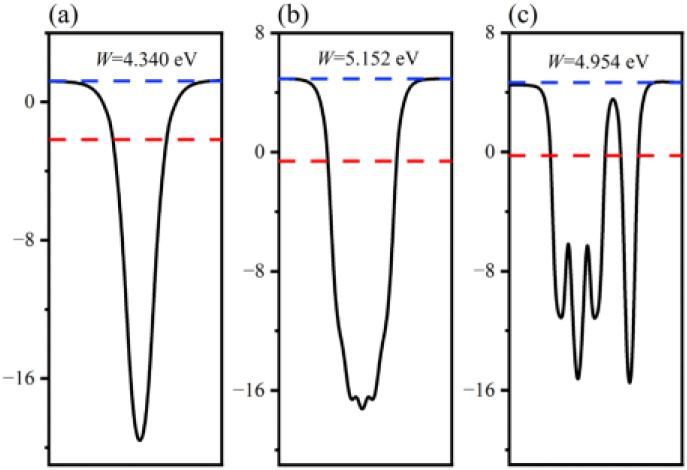
Calculated surface work functions of: (a) GaN monolayer; (b) WSe_2_ monolayer; and (c) GaN/WSe_2_ heterostructure.

The charge transfer phenomenon at the heterostructure interface can be illuminated by calculating the interfacial work function of the GaN monolayer, WSe_2_ monolayer and GaN/WSe_2_ heterostructures.^46^ The calculation formula is as follows:

4 *W* = *E*_vac_ − *E*_F_

where *E*_vac_ and *E*_F_ express the vacuum energy level and the Fermi energy level, respectively, and the vacuum energy level is taken as 4.5 eV. The GaN/WSe_2_ heterostructure curve is the sum of the GaN monolayer and WSe_2_ monolayer. The surface work functions of the GaN monolayer (Fig. 4), WSe_2_ monolayer and GaN/WSe_2_ heterostructure are 4.340 eV, 5.125 eV and 4.954 eV, respectively, and the work function of the heterostructure lies between that of isolated GaN and WSe_2_. and the electrons usually flow from the low side of the work function to the high side. Therefore, when the WSe_2_ and GaN monolayers are in contact, electrons aggregate at the interface of the WSe_2_ side to form a negative region, and the holes accumulate at the GaN side to form a positive region, thereby forming a built-in electric field pointing from the GaN layer to the WSe_2_ monolayer. Given that the Fermi levels of the two monolayers are different, the energy bands shift accordingly until the Fermi levels of the two monolayers reach equilibrium. The presence of a built-in electric field can improve the mobility of carriers, thereby enhancing the dissociated efficiency of photo-induced electron–hole pairs, reducing the recombination probability of carriers, and improving the photocatalytic properties of the heterostructure.

**Fig. 5:**
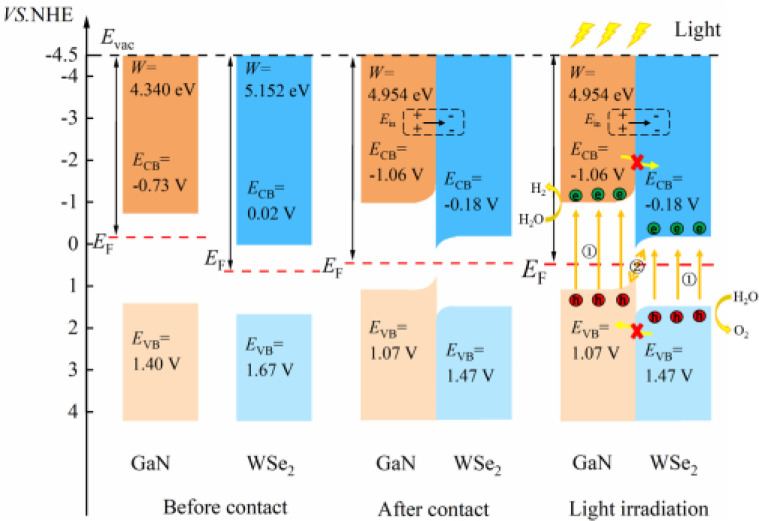
Electronic energy band alignments for isolated GaN and WSe_2_ before contact, after contact, and under illumination, displaying the segregation and transformation mechanisms of photoinduced charges.

The position of the band edge of a semiconductor is very important for evaluating its redox ability and photocatalytic performance. It is apparent that with the emergence of heterostructures, the *E*_F_ of two pristine materials will achieve equilibrium. Consequently, the isolated GaN intimately contacts with WSe_2_, which will provoke a negative shift of 0.614 V in *E*_F_ for GaN and induce a positive shift of 0.171 V for the WSe_2_ nano-slab. Due to the migration of electrons from monolayer GaN to WSe_2_ with abandoned holes in the GaN nanolayer, the edge potentials in the CB and VB of GaN are −0.73 V and 1.40 V at the GaN/WSe_2_ heterostructure after equalizing, singly. Meanwhile, that of the WSe_2_ slab is 0.02 V and 1.67 V, respectively. It can be deduced that the VB edge potential of WSe_2_ is 0.27 V lower than that of monolayer GaN; meantime, the CB edge of GaN is 0.75 V larger than that of the WSe_2_ slab. As shown in Fig. 5, the photogenerated carriers obey two different routes, as follows: (1) electron transformation: on the one hand, the photogenerated electrons transfer from the VB to the CB under illumination. On the other hand, the built-in electric field of the interface prevents electrons from migrating from the isolated GaN to WSe_2_. Meanwhile, the occurrence of CBO impedes electrons implanting in the CB of the monolayer WSe_2_, and the photo-induced holes in the VB of the single WSe_2_ hinder their transitions to the VB of the GaN nanosheet. (2) Recombination of charges: in the CB of isolated WSe_2_, the photogenerated electrons will rapidly recombine with the photogenerated holes in the VB of the isolated GaN, by virtue of the close range of charge conveyance between the WSe_2_ and GaN layer. Accordingly, photogenerated carriers can excellently detach after the construction of the heterostructure. Finally, it was determined that the GaN/WSe_2_ heterostructure is a representative direct Z-scheme semiconductor, which is consistent with the consequences of band alignment and DOS. The type of band arrangement can make electron–hole pairs separate effectively and diminish the recombination of carriers, increase the lifetime of minority carriers, and simultaneously retain the redox ability of the internal carriers, which is further enhanced compared to the traditional type-II heterostructure photocatalytic properties.

The two important potentials for water splitting are the O_2_/H_2_O electrode potential of 1.23 V, which can generate oxygen, and the standard hydrogen electrode H^+^/H_2_O potential of 0 V. Given that the GaN/WSe_2_ heterostructure is a Z-type heterostructure, the potential at the CB position of the heterostructure is −1.06 eV and the VB position is 1.47 eV. Comparing the water splitting potential, it can be known that the energy at the CBM in the GaN/WSe_2_ heterostructure is more negative than that of the standard hydrogen electrode (H^+^/H_2_O), and the VBM is more positive than that of the O_2_/H_2_O electrode. The BG of the GaN/WSe_2_ heterostructure is larger than the potential required for water splitting, and thus it can be deduced that the GaN/WSe_2_ heterostructure can be applied for either the hydrogen evolution reaction (HER) or oxygen evolution reaction (OER), making it a good photocatalytic heterostructure.

The formation of a heterostructure will induce interactions between the interfaces, leading to the transfer and redistribution of charges at the interface. This can be studied by calculating the differential charge density of the heterostructure. The plane differential density is defined as Δ*ρ*, which can be calculated using the following formula:

5 Δ*ρ* = *ρ*_GaN_/_WSe_2__ − *ρ*_GaN_−*ρ*_WSe_2__

where *ρ*_GaN/WSe_2__ represents the charge density of the entire GaN/WSe_2_ heterostructure system, and *ρ*_GaN_ and *ρ*_WSe_2__represent the charge density of the GaN monolayer and WSe_2_ monolayer, respectively. The plane charge differential density curve and three-dimensional charge differential density map in the *Z* direction of the GaN/WSe_2_ heterostructure were obtained, as shown in Fig. 5.

**Fig. 6:**
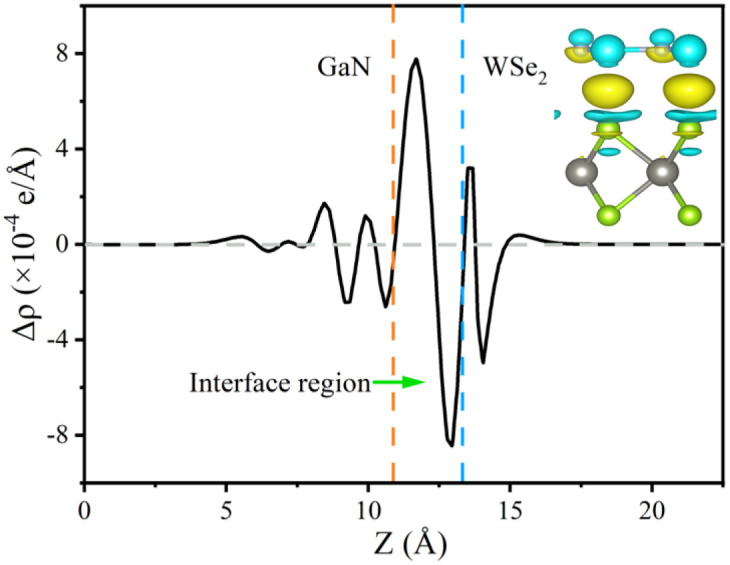
Plane-averaged and 3D charge density difference of GaN/WSe_2_ heterostructures calculated using the PBE method. The inset shows a side view of the charge density difference of the GaN/WSe_2_ heterostructure with the isosurface value of 0.0005 e Bohr^−3^.

For the plane charge differential density curve in Fig. 5, the blue area represents the depletion of charges and the yellow area represents the charge accumulation. Electrons accumulate in the WSe_2_ slab and are consumed in the GaN slab, elucidating that electrons migrate from the GaN to WSe_2_ nanosheet. To quantitatively articulate the charge density, the Bader charge analysis under equilibrium was executed. The negative charges of 0.016 e per atom shift from the GaN nanosheet to the WSe_2_ after they contact each other. It is generally acknowledged that the variation in the populations is consistent with the conclusions of the charge density difference. Simultaneously, in Fig. 5, it indicates that for the benefit of implementing stability, segments of electrons in GaN relocate to WSe_2_ in the interface, which causes a positive charge region in the surface of GaN and negative charge in that of WSe_2_. It can be confirmed that the GaN/WSe_2_ heterostructures can take advantage of the built-in electric field pointing from GaN to WSe_2_ to efficiently separate the photogenerated electrons and holes (Fig. 6).

### 3.3 Carrier mobility

**Fig. 7:**
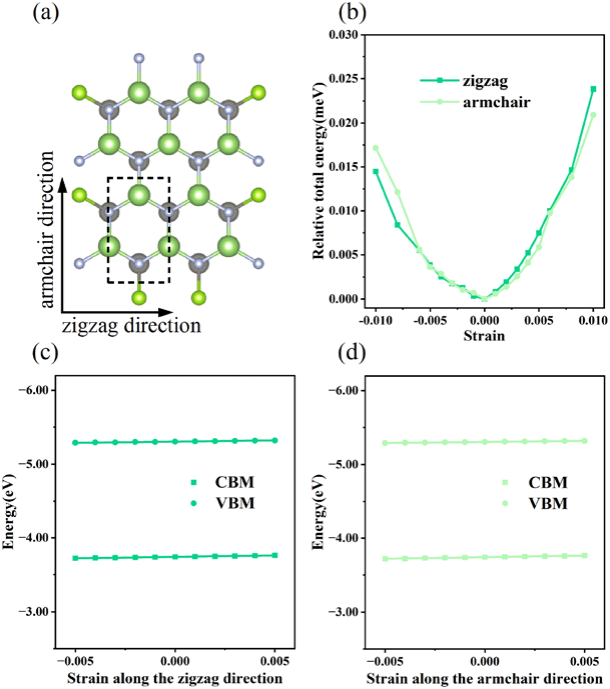
(a) Top vision of orthogonal primitive cell of GaN/WSe_2_ heterostructure. (b) Variations in relative total energy *versus* strains of GaN/WSe_2_. Tendencies of CBM and VBM *versus* strains for GaN/WSe_2_, which set *E*_vac_ as zero-point in the directions of (c) zigzag and (d) armchair. The linear curves offer the deformation potentials of GaN/WSe_2_ heterostructures.

Generally, the carrier mobility originates from the deformation potential, which is a pivotal factor to estimate photocatalytic properties and should be calculated systematically.^47^ The methods for the calculation of the carrier mobility are elaborated in the ESI.† By utilizing compressive and tensile strains, the in-plane *C*_2D_ and *E*_1_^*i*^ of the isolated GaN, WSe_2_ and GaN/WSe_2_ were assessed by fitting the data into parabolic and linear curves, which are exhibited in Fig. 7, S5 and S6 in the ESI,† respectively. Also, Table 2 displays the acquired m*, *C*_2D_, *E*_1_^*i*^ and *μ*_2D_.

**Table 2:** Carrier effective masses 
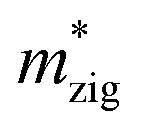
 and 
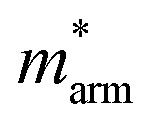
 (*m*_0_), elastic modulus *C*^zig^_2D_ and *C*^ar^_2D_m (Nm^−1^), deformation potentials *E*^zig^_1_ and *E*^arm^_1_ (eV), and carrier mobilities *μ*^zig^_2D_ and *μ*^arm^_2D_ (cm^2^ V^−1^ s^−1^) of isolated GaN, WSe_2_ and GaN/WSe_2_ under the zigzag and armchair traces at 300 K

Structure	Type	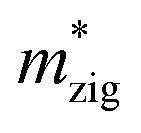	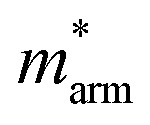	*C* ^zig^_2D_	*C* ^arm^_2D_	*E* ^zig^_1_	*E* ^arm^_1_	*μ*2Dzig	*μ*2D^arm^
GaN	Electron	1.01	1.83	256.16	279.78	−4.30	−2.46	213.93	396.91
								171 (ref. 48)	304 (ref. 49)
	Hole	1.26	1.19	256.16	279.78	−0.98	−1.26	3705.20	2574.64
								3185 (ref. 48)	2396(ref. 49)
WSe_2_	Electron	1.99	2.50	203.14	177.15	2.24	−3.26	193.79	63.84
								142 (ref. 50)	
	Hole	1.79	2.52	203.14	177.15	1.49	−2.40	511.96	122.25
								583 (ref. 51)	
GaN/WSe_2_	Electron	0.20	0.25	318.04	331.43	−5.99	−5.38	4149.37	4328.33
	Hole	0.54	0.61	318.04	331.43	−3.22	−2.91	2101.71	2395.94

The monolayer GaN and WSe_2_ and GaN/WSe_2_ heterostructure exhibit different *C*^zig^_2D_ and *C*^arm^_2D_ in the zigzag and armchair directions, which determines that their physical stress responses are totally anisotropic. In addition, the carrier mobilities of the WSe_2_ monolayer is lower than that of nanosheet GaN because of its smaller elastic modulus and higher deformation potential. These conclusions are in accordance with previous theoretical and experimental results.^48–51^ Alternatively, the limitations of the deferent rates of photogenerated carriers are mainly owing to the low electron mobilities of the GaN slab and low carrier mobilities of the WSe_2_ slab, essentially increasing the recombination of photogenerated electron–hole pairs. Monolayer GaN and WSe_2_ detrimentally obstruct the photocatalytic activities, and thus is significant to fabricate GaN/WSe_2_ heterostructures. Consequently, the electron and hole mobilities of the GaN/WSe_2_ heterostructure are 4149.37 cm^2^ V^−1^ s^−1^ and 2101.71 cm^2^ V^−1^ s^−1^ along the zigzag direction, and 4328.33 cm^2^ V^−1^ s^−1^ and 2395.94 cm^2^ V^−1^ s^−1^ along the armchair direction, respectively, indicating that the electrons in the GaN/WSe_2_ heterostructure, which have a tendency of spreading and moving along both the zigzag and armchair directions, are superior to holes. Additionally, water oxidation reactions take place in the WSe_2_ nanosheet through photogenerated holes, the water reduction reactions emerge in the GaN monolayer, as can be seen in Fig. 5. In comparison to the single layers, the electron mobilities of the GaN/WSe_2_ heterostructure in the zigzag pathway achieve a remarkable enhancement, which are 19 times and 21 times that of the isolated GaN and WSe_2_, respectively. Meanwhile, 11 times and 67 times *versus* monolayer GaN and WSe_2_ are achieved along the armchair pathway. Correspondingly, the hole mobilities of the GaN/WSe_2_ heterostructure in the zigzag direction and armchair direction are improved by 4 times and 19 times compared with that of nano-slab WSe_2_, respectively. In short, GaN/WSe_2_ heterostructures with noticeable carrier mobilities exhibit tremendous potential for application in the photocatalytic field.

### 3.4 Photocatalytic performance

To additionally validate if water splitting reactions will initiate spontaneously, the thermodynamic practicability of applying the GaN/WSe_2_ heterostructures as photocatalysts was explored. The complete water splitting mechanism of the GaN/WSe_2_ heterostructure under light irradiation was divided into the HER and oxygen OER. Herein, the Gibbs free energy (Δ*G*) of the water splitting reaction were obtained from the report by Nørskov *et al.*^52^ The method for the calculations of Δ*G* is elaborated in the ESI.† Acid (pH = 0) and neutral (pH = 7) conditions were both considered. Universally, the HER tends to occur under a suitable potential than the OER, and thus investigating the thermodynamic driving force for the OER is sufficient.

**Fig. 8:**
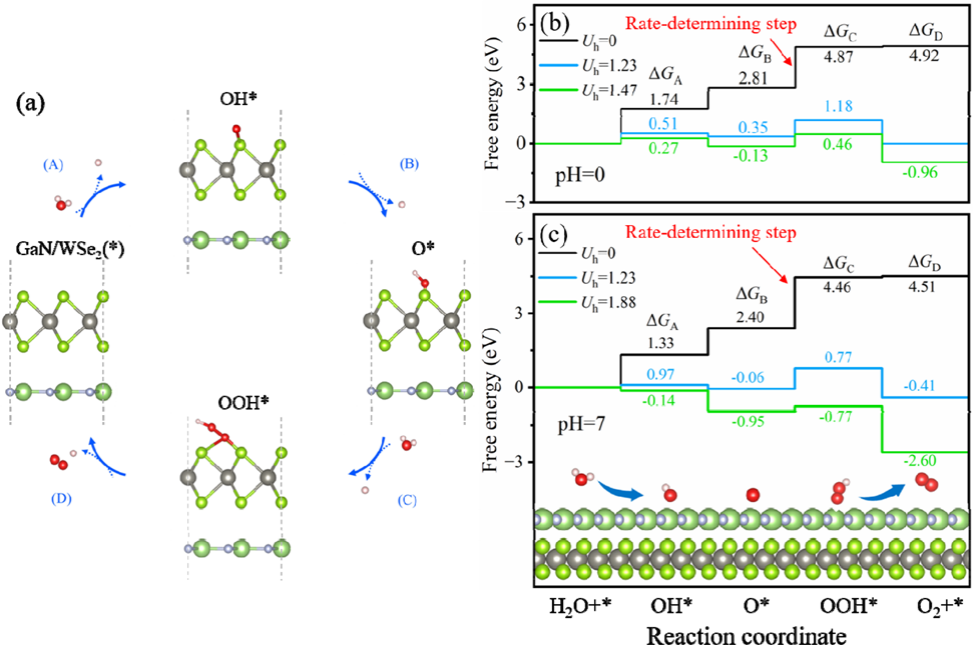
(a) Four-electron route of the OER with the rapid production of OH*, O* and OOH* intermediates on the WSe_2_ side of the heterostructures. Computational Δ*G* for the four-electron route of the OER pathway at (b) pH = 0 and (c) pH = 7.

When the OER reaction happens, the structures of the most stable OH*, O*, and OOH* intermediates adsorbed at the WSe_2_ interface during the standard four-electron transport reaction paths and the relative variation in Δ*G* are elucidated in Fig. 8. The computed Δ*G* of OH*, O*, and OOH* in the dark (*U*_h_ = 0 V) when pH = 0 is 1.74 eV, 2.81 eV and 4.87 eV, respectively. Therefore, the emergence of the OOH* intermediate is the rate-determining process, and thus the OER process can proceed spontaneously when providing a 1.47 eV external potential afforded by photogenerated holes. It is apparent that the computed Δ*G* is enhanced in the initial and third steps, but decreases in the second and fourth reaction when pH = 0 and *U*_h_ = 1.23 V.

Interestingly, when the external potential realizes 1.67 V with pH = 0, the various trends of Δ*G* are the same as the conditions at pH = 0 and *U*_h_ = 1.23 V. The results indicate that the OER step of water splitting will not spontaneously originate at pH = 0. Until the external potential reaches 1.88 V at pH = 7, the Δ*G* of the ultimate processes (Δ*G*_A_, Δ*G*_B_, Δ*G*_C_, and Δ*G*_D_) shifts to negative values, as exhibited in Fig. 8(c) (presented by the green line), which ascertains that the OER process will spontaneously occur at pH = 7 with light illumination. In conclusion, the GaN/WSe_2_ heterostructures can initiate water decomposition without a thermodynamic driving force under irradiation in a neutral environment.

## 4. Conclusions

In summary, we comprehensively investigated the electronic, optical and photocatalytic properties of the GaN/WSe_2_ heterostructure. Model V manifested a beneficial Z-scheme band arrangement with a built-in electric field pointing from GaN to WSe_2_, while model VI displayed a detrimental type I band alignment. In the case of model V, the electron mobilities of the GaN/WSe_2_ heterostructure are significantly enhanced compared to that of monolayer GaN and WSe_2_, and the hole mobilities enlarge substantially compared to that of isolated WSe_2_. In addition, the CB edge of GaN and VB edge of the WSe_2_ monolayer can yield robust reduction and oxidation reaction, respectively. Then, we explored the photocatalytic OER on the heterostructure and found that the reaction spontaneously proceeded in a neutral environment. These findings provide a theoretical basis for the practical application of 2D GaN/WSe_2_ heterostructures and provide insight for subsequent research on van der Waals heterostructures.

## Conflicts of interest

The authors declare no competing financial interest.

## Supplementary Material

RA-013-D3RA00928A-s001
