# Supplementary material for: Direct Z-scheme GaN/WSe2 heterostructure for enhanced photocatalytic water splitting under visible spectrum
Source: RSC Adv. 2023 Jul 4;13(29):20179–86. doi: 10.1039/d3ra00928a (PMC10319085; doi:10.1039/d3ra00928a)
Supplement: RA-013-D3RA00928A-s001 [file RA-013-D3RA00928A-s001.pdf]

Electronic Supplementary Material (ESI) for RSC Advances.
This journal is © The Royal Society of Chemistry 2023

Supplementary material

Direct Z-scheme GaN/WSe_2 heterostructure for enhanced photocatalytic water splitting under visible spectrum

‡ Xiaojun Ye, ‡[a] Fangfang Zhuang, [a] Yuhan Si, [a] Jingwen He, [a] Yifan Xue, [a] Hongbo Li, [b] Kai Wang, *[a] Guoqiang Hao, *[a] Rui Zhang

‡ Co-first authors: These authors contributed equally.

[a] School of Materials Science and Engineering, East China University of Science and Technology, Shanghai, 200237, China

[b] Shanghai Institute of Space Power-sources / State Key Laboratory of Space Power-sources Technology, Shanghai, 200245, China

* Corresponding Authors: Guoqiang Hao: E-mail address: haoguoqiang@ecust.edu.cn
Rui Zhang: E-mail address: zhangrui118@ecust.edu.cn

1. Calculations of model Ⅵ of GaN/WSe_2 heterostructure with DET-TS.

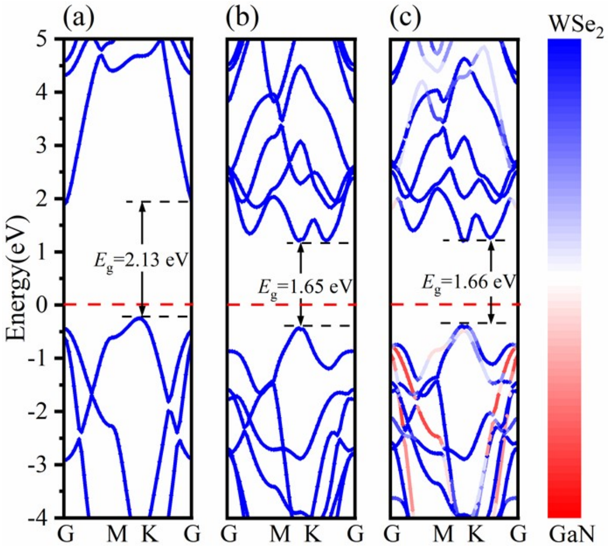

Fig. S1 Band structures of (a) isolated GaN, (b) isolated WSe_2, (c) model Ⅵ of GaN/WSe_2 heterostructure.

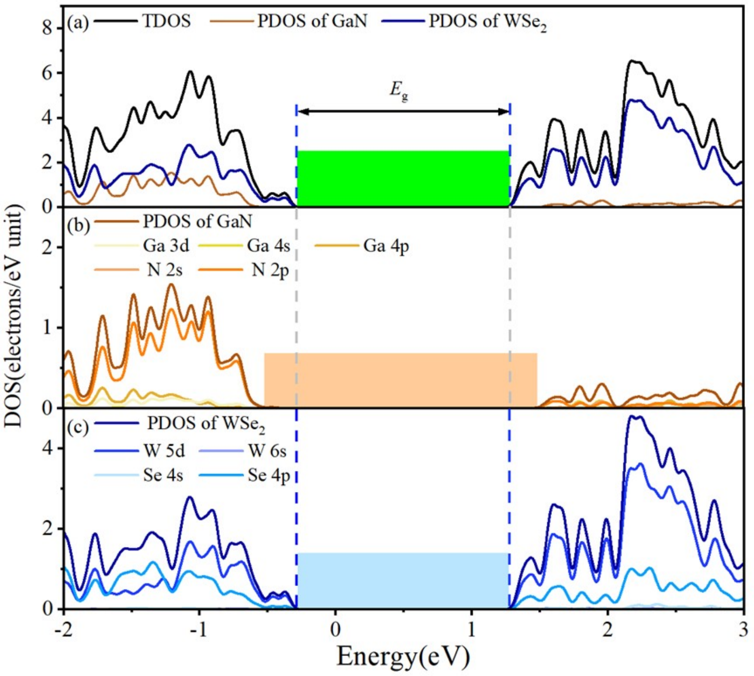

Fig. S2 (a) The calculated TDOS of GaN/WSe_2 heterostructure and PDOS of GaN and WSe_2 using DFT-TS functional. (b) The calculated PDOS of Ga, N atoms in GaN. (c) The calculated PDOS of W, Se atoms in WSe_2.

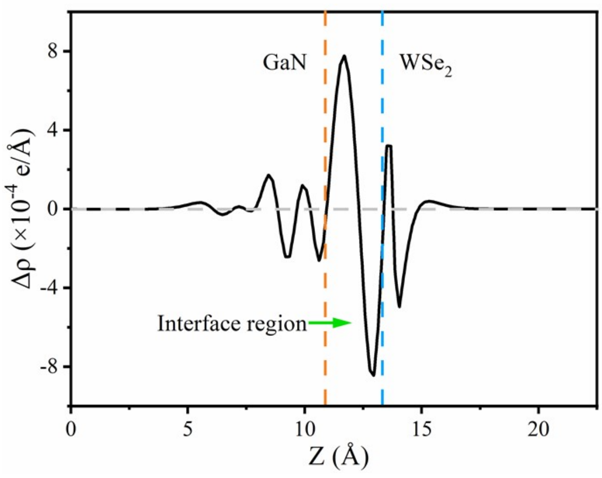

Fig. S3 Planar-averaged electron density difference Δρ(z) for GaN/WSe_2 heterostructure.

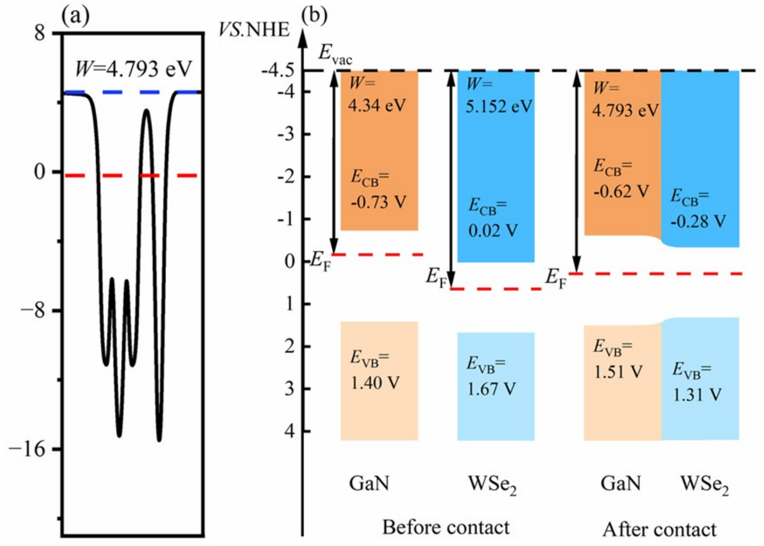

Fig. S4 Electrostatic potentials of (a) GaN/WSe_2 heterostructures of model Ⅵ. (b) Electronic energy band line-up diagrams for GaN and WSe_2 before contact, GaN and WSe_2 after contact in dark.

2. Definition of CB and VB edge potentials

The band edge energies of CB and VB of semiconductor are defined by the expression:

$$E_{\mathrm{CB}} = \chi - E_0 - 0.5E_{\mathrm{g}} \tag{S1}$$

$$E_{\mathrm{VB}} = \chi - E_0 + 0.5E_{\mathrm{g}} \tag{S2}$$

where χ is the geometric mean of absolute electronegativity of atoms. E_0 is the energy of free electron on the hydrogen scale (4.50 eV). $E_{\mathrm{C}}\sqrt{b^2 - 4ac}$ and E_{V} are the band edge energies of the CB and VB of semiconductor, respectively. E_{g} is the band gap of

semiconductors.

3. Calculations of carrier mobility

The carrier mobility can be calculated by the following formular [1]:

$$\mu_{2D} = \frac{e\hbar^3 C_{2D}}{k_B T m^* m_d \left(E_1^i\right)^2} \tag{S3}$$

where e presents the electron charge, k_B presents the Boltzmann constant and $\hbar$ represents the simplified Planck constant. The effective mass m^* is expressed as $\frac{1}{m^*} = \frac{1}{\hbar^2}\frac{\partial^2 E(k)}{k^2}$, where k represents the wave vector, $E(k)$ is the dispersion relation corresponding to k near the Fermi level. The temperature T is set to 300 K. The equivalent mass m_d is expressed as $m_d = \sqrt{m_{zig}^* m_{arm}^*}$, where m_{zig}^* and m_{arm}^* are the effective mass in the zigzag and armchair direction of the crystal, respectively. The elastic modulus C_{2D} is given by $C_{2D} = \frac{1}{S_0}\frac{\partial^2 E}{\partial \delta^2}$, where E means the total energy of the material, δ is the strain and S_0 is the surface area of the structure. The deformation potential E_1^i is determined by $E_1^i = \frac{\Delta V_i}{\Delta l / l_0}$, where ΔV_i is the energy change of the ith band with compressive and tensile strain, l_0 is the lattice length along the transfer direction and Δl is the variation of l_0 ($\Delta l / l_0$ is less than 0.5%).

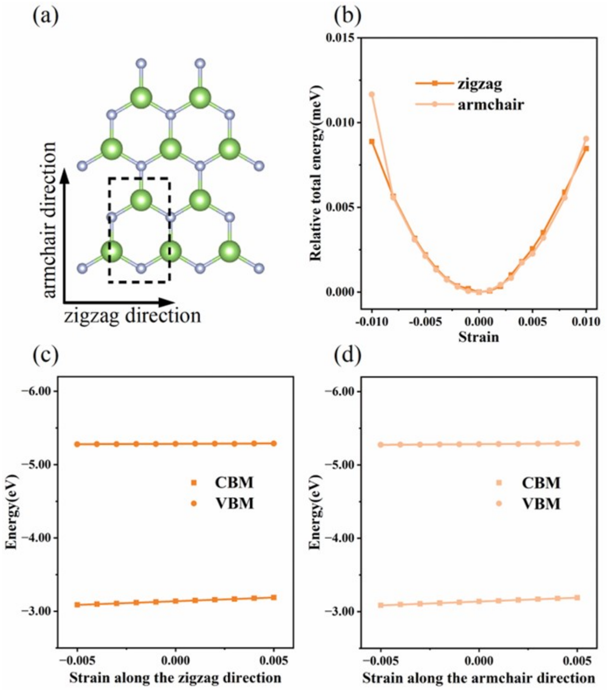

Fig. S5 (a) Top view of rectangular unit cell of GaN heterostructure. (b) The energy-strain relationship of GaN. The quadratic fitting of the data acquires the in-plane stiffness of GaN. The shift of CBM and VBM for GaN with reference to E_{vac} as a function of the utilized strain in the (c) zigzag and (d) armchair directions, respectively. The linear fitting provides the deformation potentials of GaN heterostructure.

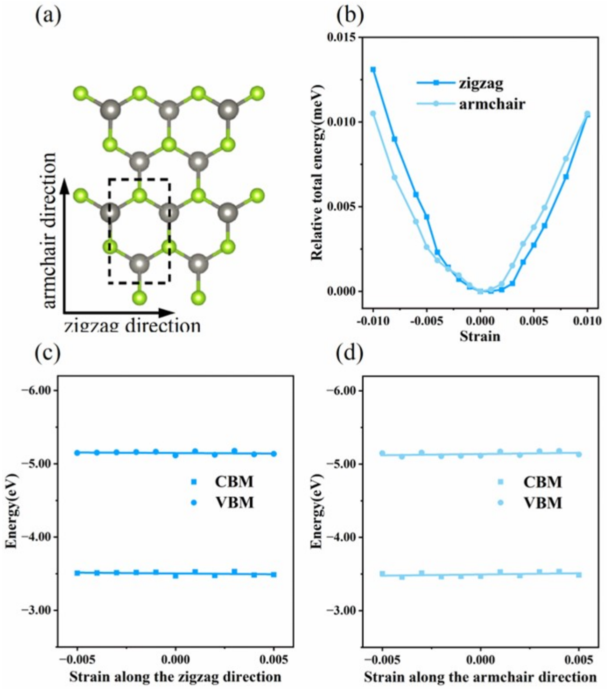

Fig. S6 (a) Top view of rectangular unit cell of WSe_2 heterostructure. (b) The energy-strain relationship of WSe_2. The quadratic fitting of the data acquires the in-plane stiffness of WSe_2. The shift of CBM and VBM for WSe_2 with reference to E_{vac} as a function of the utilized strain

in the (c) zigzag and (d) armchair directions, respectively. The linear fitting provides the deformation potentials of WSe_2 heterostructure.

4. Calculations of Gibbs free energy

The Gibbs free energy can be expressed as follow [2]:

$$\Delta G = \Delta E + \Delta E_{\mathrm{ZPE}} - T\Delta S + \Delta G_{\mathrm{U}} + \Delta G_{\mathrm{pH}} \tag{S4}$$

where ΔG presents the whole energy difference among the heterostructure and several terminations calculated by DFT-PBE method. The differences of zero-point energy and entropy between adsorbed states of intermediates and gap phases are represented as ΔE_{ZPE} and $T\Delta S$, singly. T means the room temperature (298.15 K). $\Delta G_{\mathrm{U}} = -eU$, whereby U expresses the electrode potential. ΔG_{pH} was corrected to the solutions acidity and alkalinity.

References

[1] S. Sun, F. Meng, H. Wang, H. Wang, Y. Ni, *J. Mater. Chem. A*, 2018, 6, 11890-11897.

[2] J.K. Nørskov, J. Rossmeisl, A. Logadottir, L. Lindqvist, J.R. Kitchin, T. Bligaard, H. Jónsson, *J. Phys. Chem. B*, 2004, 108, 17886-17892.
